# The first reported case of *Mycobacterium shinjukuense* pulmonary disease from China

**DOI:** 10.1128/asmcr.00167-25

**Published:** 2025-11-24

**Authors:** Reeti Khare, Minh-Vu H. Nguyen, Harleen Sahni

**Affiliations:** 1 Advanced Diagnostic Laboratories, National Jewish Health 2930, Denver, Colorado, USA; 2 Division of Infectious Diseases, Department of Internal Medicine, University of California, Davis Health 70083 , Sacramento, California, USA; 3 Division of Infectious Diseases, Department of Medicine, Santa Clara Valley Medical Center 14454, San Jose, California, USA; Rush University Medical Center, Chicago, Illinois, USA

**Keywords:** *Mycobacterium shinjukuense*, nontuberculous mycobacteria, NTM-PD, chronic pulmonary disease

## Abstract

**Background:**

This report describes the first clinical case of *Mycobacterium shinjukuense* pulmonary disease (PD) originating in China, thereby extending the known geographic distribution of this nontuberculous mycobacterial (NTM) species beyond Japan and Korea.

**Case Summary:**

*M. shinjukuense* caused chronic nodular-bronchiectatic PD in a 55-year-old woman. Computed tomography scans demonstrated persistent nodules and bronchiectasis over an 8-year period (2018–2025). A definitive diagnosis was achieved via a positive bronchoalveolar lavage culture followed by subsequent repeated detection in sputa 3 years after the initial presentation. Her symptoms were productive cough, hemoptysis, and dyspnea, and they fluctuated throughout this time, as did culture positivity on serial sputum testing. She improved clinically on airway clearance therapy alone and did not need antimicrobial therapy.

**Conclusion:**

This case highlights the indolent progression and diagnostic delay often associated with NTM-PD, as well as the presence of *M. shinjukuense* in new geographic regions.

## INTRODUCTION

Nontuberculous mycobacteria (NTM) are increasingly recognized as important causes of pulmonary disease (PD), particularly in patients with underlying conditions such as bronchiectasis (1). Among them, *Mycobacterium shinjukuense* is an extremely rare, slowly growing, non-chromogenic species found primarily in Japan, with one case described from Korea (2–4). Its close phylogenetic relationship to the *M. tuberculosis* complex (MTBC) may cause cross-reaction with some molecular assays designed for tuberculosis and potentially lead to misdiagnosis (2, 4–7). Clinical manifestations include nodular and cavitary lung disease, but optimal management strategies remain undefined (4, 7). Here, we present the first reported case of *M. shinjukuense* PD originating in China, describing the patient’s prolonged disease course, diagnostic evaluation across multiple international centers, and long-term management primarily with airway clearance therapy in the absence of directed antimicrobial treatment.

## CASE PRESENTATION

**Fig 1:**
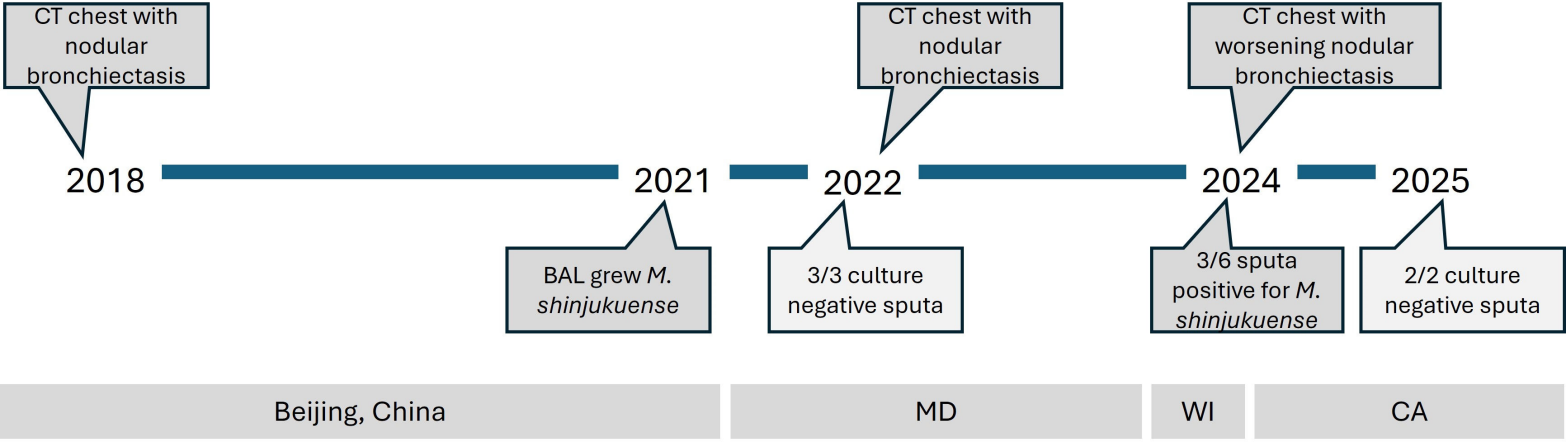
Timeline of known diagnostic workup. CT = computed tomography, MD = Maryland, WI = Wisconsin, CA = California.

A 55-year-old woman, with a history of hypothyroidism and a body mass index of 21, was born and raised in Liaoning Province, China. She moved to Beijing and underwent a chest computed tomography (CT) scan in 2018 as part of a routine physical exam offered by her company and was found to have pulmonary nodules and bronchiectasis (Fig. 1). At that time, she did not have any chronic respiratory symptoms, no prior history of tuberculosis infection, recurrent pneumonias, gastroesophageal reflux disease, or hospitalizations. She was prescribed a 2-week course of azithromycin and a cephalosporin, but there was no apparent change on subsequent CT scans in 2019 and 2020. She worked in finance for an oil company, has been a lifelong non-smoker and non-drinker, and reported no travel outside of China.

In February 2021, she underwent a bronchoscopy in China, and a mycobacterial culture from the bronchoalveolar lavage grew a slowly growing mycobacterium that was identified as *M. shinjukuense* (unknown methodology). At that time, she had a chronic productive cough and occasional scant hemoptysis. The patient was placed on a multidrug regimen of amoxicillin, clarithromycin, ofloxacin, and ethambutol, but all medications were discontinued after one month due to drug intolerability. In December 2021, the patient immigrated to Baltimore, Maryland, US and was evaluated by a pulmonologist. She continued to have a productive cough, occasional blood-tinged sputum, with no dyspnea or other systemic symptoms.

**Fig 2:**
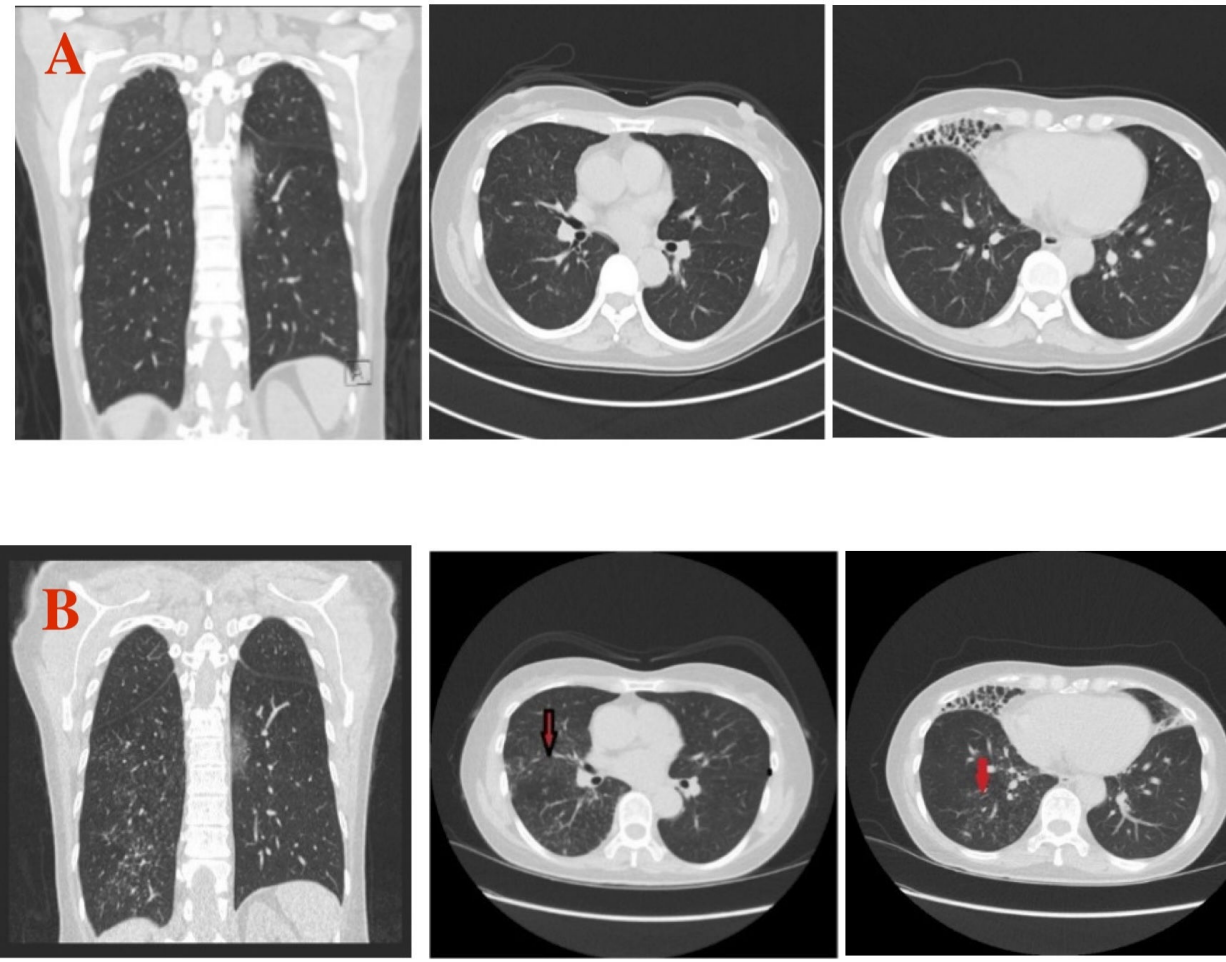
Features of *M. shinjukuense*. (**A**) Chest CT scan from April 2022. Left: coronal view. Middle: transverse view with mild nodularity in the right upper lobe. Right: bronchiectasis in the right middle lobe and mild nodularity in the right lower lobe. (**B**) Chest CT scan from August 2024. Left: worsening nodularity seen in the right middle and lower lobes. Middle: worse nodularity in the right upper lobe. Right: worse nodularity in the right lower lobe.

Repeated CT scans in 2022 continued to show stable tree-in-bud opacities and bronchiectasis in the right middle lobe, lingula, and right lower lobe consistent with NTM-PD (Fig. 2). She underwent comprehensive testing to look for other causes of bronchiectasis, including antinuclear antibodies, rheumatoid factor, connective tissue disease serologies (anti-SSA and SSB antibodies), immunoglobulin levels, and *Aspergillus* antibodies, which were all negative. She was HIV negative. Airway clearance therapy with 3% hypertonic saline and an oscillating positive expiratory pressure (OPEP) device (Aerobika, Monaghan Medical Corporation) was initiated twice a day, which improved the productive cough over time. Three expectorated cultures taken from April through June 2022 did not grow any mycobacteria (Fig. 1).

She was then relocated to Wisconsin in early 2024, where two out of three sputum samples were acid fast bacilli (AFB) smear and culture positive for *M. shinjukuense*, as identified by 16S rDNA sequencing (Wisconsin State Lab of Hygiene, Madison, WI) and *rpoB* sequencing (Mycobacteriology Lab, National Jewish Health, Denver, CO) (Fig. 1). Antimicrobial susceptibility testing could not be completed because of insufficient growth of the organism. The remaining culture was positive for *Nocardia nova*, which was considered not clinically significant. The patient was hesitant to start any antimicrobial treatment for NTM, and therefore, airway clearance therapy was continued.

In August of 2024, she had a small amount of ongoing productive cough but overall described clinical improvement and was able to run 4 miles at a slow pace without being short of breath. However, a repeated chest CT scan showed increased lower lobe bronchiolocentric nodules and severe middle lobe bronchiectasis (Fig. 2). One out of three cultures in October 2024 was still positive for *M. shinjukuense*. Her productive cough continued into January 2025, but two out of two sputum cultures were negative (Fig. 1). Susceptibility testing for the isolate from October 2024 was performed using broth microdilution, with breakpoints (susceptible = S, resistant = R, no interpretation = NI) as recommended by the Clinical Laboratory Standards Institute for slowly growing mycobacteria (8). Minimum inhibitory concentrations were as follows: amikacin, 16 (S); ciprofloxacin, >8 (R); clarithromycin, 0.25 (S); clofazimine, ≤0.015 (NI); doxycycline, 8 (R); linezolid, 2 (S); minocycline, 8 (R); moxifloxacin, 2 (I); rifabutin, ≤0.12 (S); rifampin, 0.25 (S); streptomycin, 32 (NI); and trimethoprim/sulfamethoxazole, ≤0.3/4.8 (S). However, she remained clinically stable and chose to continue with airway clearance therapy only.

## DISCUSSION

This case study describes the first patient with *M. shinjukuense* PD that originated from China in current literature. She met diagnostic criteria for nodular-bronchiectatic PD based on her compatible symptoms of worsening nodules, bronchiectasis, and repeated detection of the same NTM species from multiple respiratory specimens (9). Despite waxing and waning symptoms, her CT scans showed progression of disease over years, which is characteristic of NTM PD.

**Fig 3:**
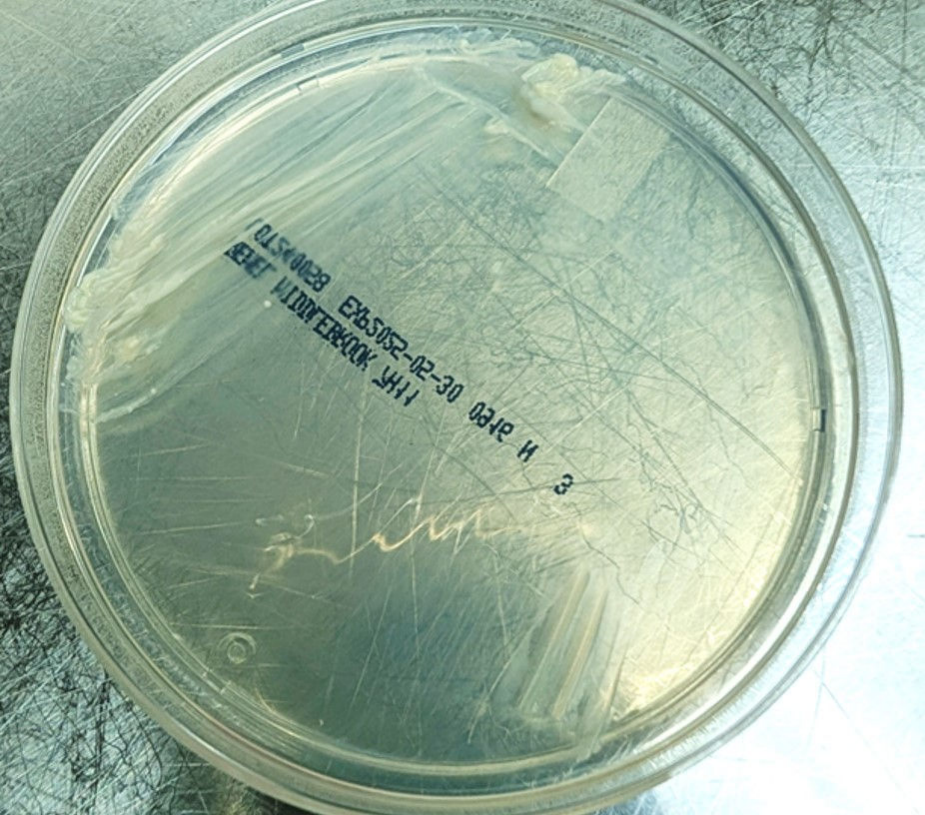
Growth on 7H11 agar at 37°C 22 days after subculture.

*M. shinjukuense* is a rare cause of nodular-bronchiectatic or cavitary PD. It is a slowly growing, non-chromogenic NTM that may grow at temperatures of 30–37°C without distinguishing characteristics (Fig. 3) (2). Its ecological niche is not yet known, but like other NTM, it is likely acquired through exposure to soil or water. *M. shinjukuense* was named after the Shinjuku ward of Tokyo in 2011, and only ~50 previous cases have been published, all occurring in Japan or Korea (2, 4). In this case, the patient’s infection originated in Northeastern China, with no known travel history to Japan or Korea. This marks a deviation from the organism’s established geographic distribution.

Phylogenetic analysis shows that this organism has high sequence similarity to MTBC when using the 16S rRNA gene (2). It shares several features with MTBC and is only one of four NTM that form a common clade called the Mtb-associated phylotype (5). As a result, it may cross-react with some assays, such as the TRCRapid M.TB (Tosoh Bioscience, Tokyo, Japan), DNA probe FD-MTD (Fujirebio Inc., Tokyo, Japan), and possibly the TSPOT.TB (Revvity), and may be mistakenly diagnosed as MTBC; this may confound appropriate treatment (2, 4, 6, 7). To note, a GeneXpert MTB/Rif assay (Cepheid, Sunnyvale, US) was performed on this patient in 2024 that was negative. Assays performed on isolates, such as 16S rRNA, *rpoB*, hsp65, 16S-23S ITS sequencing, and MALDI-TOF mass spectrometry, were able to correctly identify it (2, 10).

A recent study from Japan found that the clinical relevance of *M. shinjukuense* was high, with 100% (7/7 across a 5 year time period) of cases identified in samples being associated with PD based on clinical assessment and radiological analysis (3). No treatment guidelines exist for this organism, but it has been successfully treated in previous cases with standard *M. avium* complex (MAC) regimens, such as rifampin, ethambutol, and a macrolide (4, 10). *In vitro* susceptibility testing in prior reports showed susceptibility to these antibiotics (10). Interestingly, clinical improvement was also seen in cases treated with regimens considered unsuitable for NTM, such as MTBC regimens (i.e., use of rifampin, ethambutol, isoniazid, and no macrolide) (6, 7, 10). Many NTM (excluding those such as *M. kansasii*) are typically considered intrinsically resistant to isoniazid (11), but *M. shinjukuense* was previously found to be susceptible to isoniazid (as well as susceptible to rifampin and ethambutol), possibly because of its genetic similarity to MTBC (5). Research testing of this patient’s isolate by agar proportion also showed susceptibility to isoniazid (and rifampin and ethambutol). Despite available treatment regimens, it is recognized by experts that the first step in treating NTM-PD is to treat the underlying chronic lung disease, such as our patient’s bronchiectasis, for which airway clearance therapy is recommended (1). As a case in point, this patient managed her disease using airway clearance therapy for 4 years without NTM-directed antibiotics, though these may be needed in the future given radiologic progression.

### Conclusion

This case expands the known geographic range of *M. shinjukuense* to include areas of China. It also underscores the potential for significant diagnostic delay in NTM infections, which may extend over several years (12). Notably, despite the prolonged course of disease, a range of management approaches—including standard MAC therapy, first-line antituberculous therapy, or airway clearance therapy without antimicrobial management—may result in clinical improvement (10). These findings reflect our incomplete understanding of the optimal treatment strategies for *M. shinjukuense* and highlight the need for further research into its natural history and management.

## AFTER EPUB

*[This article was published on 24 November 2025 with a typographical error in the abstract. The abstract was updated in the current version, posted on 13 February 2026.]*
